# Young carers’ mental health during and after the Covid-19 pandemic: variation by gender, ethnicity, income and peer, school or social support

**DOI:** 10.1007/s00127-026-03111-6

**Published:** 2026-05-19

**Authors:** Rebecca E Lacey, Alejandra Letelier, Beth Neale, Andy McGowan, Steven Guan, Anne McMunn

**Affiliations:** 1 School of Health & Medical Sciences, City St George’s, University of London, Cranmer Terrace, London, SW17 0RE UK; 2 Research Department of Epidemiology and Public Health, UCL, London, UK; 3 Carers Trust, London, UK

**Keywords:** young carer, depression, Covid-19, anxiety, mental health, inequality

## Abstract

**Purpose:**

Young carers are an often overlooked group of carers, with little longitudinal research on their mental health, particularly regarding variations across demographics and support systems. This study examined whether young carers have poorer mental health than peers and explored inequalities by gender, ethnicity, special educational needs (SEN) status, income, parental composition, and support systems.

**Methods:**

We used the COVID Social Mobility and Opportunities cohort study, 2021–2023, a probability sample of > 13,000 young people aged 16–18 in England. We assessed self-reported young caring and mental health outcomes, including psychological distress, anxiety, depression and chronic mental illness. Modifiers were ethnicity, gender, household income, special educational needs (SEN) status, parental composition, perceived school support for mental health, peer support and social support.

**Results:**

Young carers reported 1.69 higher odds of chronic mental illness (95% CI: 1.35, 2.12), and more depressive (B = 0.20, 95% CI: 0.07, 0.33) and anxiety symptoms (B = 0.15, 95% CI: 0.02, 0.29). Associations did not vary by gender, ethnicity, SEN status, parental composition or income. No statistical differences were observed by peer, social or school support.

**Conclusions:**

Young carers had poorer mental health which did not vary across key axes of inequality. Addressing their mental health is crucial to prevent a cycle of care need. Increasing societal awareness and fostering empathy and support may help improve young carers’ mental health.

**Supplementary Information:**

The online version contains supplementary material available at https://doi.org/10.1007/s00127-026-03111-6.

## Introduction

Young carers are young people aged < 18 years who provide help or support to a family member or friend because of a physical or mental illness, disability, frailty, or addiction [1]. This care is often provided to a parent, sibling, or grandparent [2, 3], but can also include friends where activities and support go beyond what would usually be expected as friendship at this age. The prevalence of young carers is challenging to estimate, as young carers are often hidden from services due to fear of social services involvement or being unaware of their caregiving role. In the US, young carers have only recently been recognised, and recent estimates suggest that more than 5.4 million children and young people < 18 years (~ 7.5%) are young carers [3]). In the UK, where there has been much greater and longer-term recognition of young carers [4], estimates from the 2021 Census for England and Wales suggest that there were 120,000 young carers aged < 18 years (~ 1.4%, 28,000 in Scotland [5]17,500 in Northern Ireland [6]); but when young people themselves were asked about whether they have caring responsibilities, the UK prevalence was more like 11% (Letelier et al., 2024).

Young caring is known to have significant impacts on a wide range of outcomes. This includes on mental and physical health [7–9], subjective wellbeing ([10, 11], education [12], and social relationships [13]. While there has been much research on young carers over the past 30 years, there have been very few quantitative longitudinal studies to date examining the mental health effects of young caring [7]. That which has been conducted covers the pre-Covid period and evidence has shown that the Covid period affected population mental health, including that of parents [14] and young people [15]. In turn, the prevalence of young caring increased and likely its potential consequences [2, 16]. The extant pre-Covid longitudinal studies found that young carers were more likely to report depression a few years later in the Longitudinal Study of Young People in England (LSYPE) [17], were more likely to report a reduction in subjective wellbeing around becoming a young carer in the UK Household Longitudinal Study (UKHLS) [10], had more psychological morbidity in the Australian Household, Income and Labour Dynamics in Australia (HILDA) survey [18], and more symptoms of psychological distress in the Longitudinal Study of Australian Children (LSAC) [8]. We do not yet know whether these findings are replicated during or after the Covid-19 pandemic. The pandemic brought unique challenges in the accessibility of usual support mechanisms (e.g. health and social care services) and this was disproportionately the case for vulnerable groups, such as children with safeguarding concerns [19]. Given that young carers are frequently “hidden in plain sight” to services, the Covid pandemic exacerbated existing challenges in young carer identification and support [20]. Young carers in England reported having to “step-up” their caring role during the pandemic to cover the gaps caused by reductions in external agency support and this has implications for their ability to meet their own mental health needs and managing remote engagement with school during lockdowns [21].

Further, there has been very little investigation of whether associations between young caring and mental health vary by axes of inequality, such as gender, income, ethnicity, parental composition, and Special Educational Needs (SEN). This study draws on Crenshaw’s intersectionality framework [22], positing that intersecting identities (e.g. between young caring and ethnicity) may shape an individual’s outcomes differently. Prior analyses of LSAC found no evidence of a gender-young carer interaction [23]. Yet this interaction may have existed during the Covid period as evidence shows that female young carers had higher care loads than male young carers during this period which may impact differently on mental health [2]. In the UK, there are also social inequalities in young caring prevalence with over-representation of young people from Pakistani or Bangladeshi ethnic groups, lower income households, single parent households and those with SEN [2]. There are also important systemic inequalities by ethnicity and socioeconomic circumstances in access to support services for both young people and adults who need external agency support e.g. health [24] and social care [25] services. We do not yet know whether associations between young caring and mental health differed by ethnicity, gender, socioeconomic position and SEN.

Finally, to inform policies and practices that better support young carers, it is important to investigate what might reduce the potential mental health impact of young caring. For adolescent young carers, the most proximal support mechanisms are those provided by school, peers and social networks. Access to the usual support mechanisms for young carers was reduced during the Covid pandemic, impacting on them being able to manage their mental health needs [21]. A recent qualitative study found that young carers valued support in schools and having an understanding source of social support [26]. However, no study has examined how these potential support factors modify the relationship between young caring and mental health. Knowing whether these factors effectively reduce the mental health impact of young caring would provide weight to funding and promoting interventions that improve these support aspects.

Therefore, this study aimed to assess the mental health of young carers compared to non-carers in a longitudinal study and whether these associations differed by gender, ethnicity, SEN, household income, parental composition during the Covid pandemic in England. We additionally sought to test whether the young persons’ perception of school, peer and social support mitigated any observed associations.

## Materials and methods

### Participants

This study used data from the Covid Social Mobility and Opportunities (COSMO) study, a cohort study of young people in England in school year 11 (aged 16–17) in 2020-21. COSMO used a multi-stage stratified cluster random probability sample from the National Pupil Database (an education administrative database containing all children and young people in English state schools) plus additional private school sampling (using the Department for Education’s Get Information About Schools database) [27]. Schools with over-representation of young people from disadvantaged backgrounds and from ethnic minorities were oversampled. This resulted in more than 500 schools and 13,787 young people plus 11,731 parents participating in wave 1 (2021-22) [27]. All young people from wave 1 were invited to take part in wave 2 (2022–2023), of whom 11,523 young people and 10,678 parents were included [28]. This study uses data from the first two waves of COSMO.

## Outcomes

For the outcome, four young person-reported mental health measures were included from waves 1 and 2: psychological distress (as indicated by the 12-item General Health Questionnaire (GHQ-12) [29] ), anxiety (2-item Generalized Anxiety Disorders Questionnaire (GAD-2) [30]), depressive symptoms (2-item Patient Health Questionnaire (PHQ-2) [31]) and chronic mental health condition (wave 2 only). The GHQ-12 items covered symptoms of psychological distress (e.g. ability to concentrate, feeling strained) in the past few weeks. Response categories were better than usual, same as usual, less than usual, and much less than usual. Individual items were summed to create a score with range 0–36 with higher scores indicating more psychological distress. Cronbach’s alpha for GHQ-12 at waves 1 and 2 was 0.92. GAD-2 asked young people to rate how often they had felt nervous, anxious and on edge, and not being able to stop or control worrying over the past two weeks (wave 1 alpha = 0.84; wave 2 alpha = 0.86). The PHQ-2 asked young people how often they had little interest or pleasure in doing things, and feeling down, depressed or hopeless over the past two weeks (wave 1 alpha = 0.77; wave 2 alpha = 0.78). Response categories for the GAD-2 and PHQ-2 were not at all, several days, more than half the days, and nearly every day. Responses were summed to create continuous variables with ranges 0–6. Reporting of a chronic mental health condition was binary (“yes” or “no”).

## Exposure

Regarding exposure, young people were asked “Do you regularly look after anyone who is ill, disabled or elderly and in need of care, without being paid? This includes people who live with you and who live elsewhere, but please don’t include volunteering” Young people answering yes were considered to be young carers, with wave 1 caring status used as the exposure to preserve temporal ordering.

## Covariates

We examined possible modification by gender, ethnicity, household income, parental composition, SEN status, plus perceived school, peer, and social support. All variables were taken from wave 1. Gender was coded as male, female or non-binary. Ethnicity was collected as 18 groups and condensed to White, Asian/Asian British, Black/African/Caribbean/Black British, Other/Mixed. Annual household income (parent-reported) was collected as 20 banded categories, and recoded to approximate quintiles as <£19,000, £19–32,500, £32,5–46,000, £46–75,000 and >£75,000. Parental composition (parent-reported) was classified as two or single-parent family. SEN status was approximated if the young person reported having attended school during Covid lockdowns because of SEN (a priority group during lockdown).

Social support was indicated via the short Social Provisions Scale (SPS-3). Three questions were: “I have family and friends who help me feel safe, secure and happy”, “There is someone I trust whom I would turn to for advice if I were having problems”, and “There is no one I feel close to”. Response categories were very true, partly true and not true at all. Responses to questions one and two were reverse coded so that each response ranged from 1 (not true at all) to 3 (very true). Responses across items were summed and then binarized as 1–6 for low support and 7–9 as high support. This was based on categorisations of the SPS-3 in other studies [32]. Peer support was indicated by the 3-item People in My Life Questionnaire. The three items in this scale were: “My friends listen to what I have to say”, “I can count on my friends to help me when I have a problem”, and “I share my thoughts and feelings with my friends”. Possible responses to all items were never true, sometimes true, often true, and always true. Total scores of 1–6 indicated low support and scores of 7–12 high support. Finally, young people were asked to rate the support their school provided for mental health and wellbeing as very good, fairly good, not very good or not at all good. Very and fairly good were collapsed to indicate good school support, and other categories as poor support.

Covariates were wave 1 mental health measures (GHQ-12, PHQ-2, GAD-2), plus school type (private vs. state), parental age (< 35 yrs, 35–39 yrs, 40–44 yrs, 45–49 yrs, 50–54 yrs, 55+) and parental highest level of education (University degree/diploma, Advanced(A)-level or equivalent (typically taken at age 18), General Certificate of Secondary Education (GCSE/CSE)/Ordinary(O)-level or equivalent (typically taken at age 16), Other, None).

### Statistical analysis

Multiple imputation by chained equations was used to impute missing information for all cohort members and analyses included those with at least one mental health outcome observed in wave 2 (*n* = 11,207) [33]. Twenty imputations were run including all analysis variables plus auxiliary variables predictive of missingness, such as area-level deprivation and copies of wave 1 covariates from wave 2. Subsequent analyses were run for those with each observed outcome [33]. 

Descriptive statistics were run for those young people with at least one mental health outcome observed (*n* = 11,207). In subsequent regression analyses those with observed information on each outcome were analysed (GHQ: *n* = 11,110; PHQ: *n* = 10,639; GAD: *n* = 10,628; chronic mental illness: *n* = 9,571). Linear regression was used to examine associations between young caring and GHQ, PHQ and GAD, firstly unadjusted, second adjusting for covariates and finally including baseline mental health. Logistic regression was used for associations between young caring and reporting a chronic mental health condition. The final model adjusting for baseline chronic mental health conditions could not be run for this outcome as this information was not available at wave 1.

Interaction terms between young caring and potential modifiers were tested. Where interactions were present, we computed the predicted mean mental health using Stata’s ‘mimrgns’ command for each caring/modifier strata and computed the contrasts to test significance of the differences. In a sensitivity analysis we ran all analyses in a complete case dataset. Survey weights were applied throughout the analyses to account for the unequal probability of selection and to compensate for systematic non-response. All analyses were conducted in Stata v18.0 [34].

The protocol for this study was pre-registered: https://osf.io/k98hw/.

## Results

### Descriptive analysis

Of the 11,207 participants with at least one observed mental health outcome, 11.6% were young carers, 51% were male, 74.6% were from two-parent families, almost three-quarters (72.6%) were from White ethnicities, and 2% had SEN (Table 1). Young people reported high levels of psychological distress, anxiety, depression and chronic mental illnesses. Average levels of psychological distress (GHQ) increased between the two waves but depressive and anxiety symptoms remained relatively stable.

Mean mental health scores were higher in young carers compared to non-carers, indicating poorer mental health. Further, more than 1 in 5 (21.1%) of young carers had a chronic mental health condition compared to 14.4% of non-carers. Most covariates differed between carers and non-carers. Carers were more likely to come from Asian/Asian British and Mixed/Other ethnicities, were more likely to come from lower income and single parent households, have younger parents and have parents with no or lower-level qualifications.

**Table 1:** Characteristics of the study sample (*n*=11,207)^a^

	Carers	Non-carers	Total
	11.60%^b^	88.40%	
	**Mean (SE)**	**Mean (SE)**	**Mean (SE)**
*W2 outcomes*			
GHQ	14.40 (0.30)	13.71 (0.10)	13.79 (0.10)
Missing (%)	0.10	0.60	0.87
GAD	2.32 (0.07)	2.00 (0.03)	2.04 (0.03)
Missing (%)	5.10	3.85	5.17
PHQ	2.05 (0.07)	1.70 (0.02)	1.73 (0.02)
Missing (%)	4.62	3.80	5.07
	**%**	**%**	**%**
Chronic mental illness (yes)	21.08	14.41	15.18
Missing	15.92	12.30	14.60
*W1 mental health*	**Mean (SE)**	**Mean (SE)**	**Mean (SE)**
GHQ	14.20 (0.11)	13.26 (0.11)	13.37 (0.11)
Missing (%)	0.31	0.26	2.03
GAD	2.37 (0.08)	1.99 (0.03)	2.03 (0.03)
Missing (%)	3.40	0.33	6.02
PHQ	2.03 (0.08)	1.71 (0.03)	1.75 (0.03)
Missing (%)	3.50	3.09	5.83
*Potential modifiers*	**%**	**%**	**%**
SEN status	2.00	2.00	2.03
Missing	0.00	0.00	0.00
Ethnicity			
White	68.04	73.17	72.58
Asian/Asian British	16.05	12.74	13.12
Black	6.89	6.25	6.33
Mixed/Other	9.01	7.84	7.97
Missing	38.8	27.89	31.76
Gender			
Male	51.46	50.97	51.03
Female	47.48	47.29	47.31
Non-binary	1.06	1.74	1.66
Missing	2.15	1.67	2.02
Household income			
<£19,000	38.67	24.48	26.12
£19-32,500	26.36	22.55	22.99
£32,500-46,000	15.82	18.12	17.85
£46-75,000	12.78	21.55	20.54
>£75,000	6.37	13.30	12.50
Missing	67.77	54.57	61.55
Parental composition			
Two parents	65.57	75.80	74.62
One parent	34.43	24.20	25.38
Missing	9.08	6.91	7.95
* Support variables*			
School support			
Poor support	49.82	46.65	47.01
Good support	50.18	53.35	52.98
Missing	4.77	6.48	9.82
Social support			
Low support	23.28	19.16	19.64
High support	76.62	80.84	80.36
Missing	0.46	0.60	2.74
Peer support			
Low support	26.06	23.16	23.49
High support	73.94	76.84	76.51
Missing	1.00	1.05	3.21
*Covariates*			
School type			
Independent	2.44	1.19	1.34
State	97.56	98.81	98.66
Missing	44.1	41.79	45.01
Parental age (years)			
<35	9.47	5.81	6.23
35-39	15.62	11.35	11.85
40-44	21.97	20.38	20.56
45-49	22.68	28.03	27.41
50-54	19.53	23.98	23.46
55+	10.73	10.45	10.48
Missing	25.00	21.25	23.51
Parental education			
None	13.79	8.84	53.26
Other	4.18	2.95	9.65
GCSE/CSE/O-level or equivalent	30.87	23.76	24.58
A-level or equivalent	9.84	9.63	3.09
University degree/diploma	41.32	54.82	9.41
Missing	15.77	12.17	14.56

^a^Descriptive statistics presented for cohort members with at least one mental health outcome observed at wave 2; ^b^Pooled percentages across imputed datasets presented as specific Ns vary across imputations.Abbreviations: A-level = Advanced level; CSE = Certificate of Secondary Education; GAD = Generalized Anxiety Disorders (anxiety symptoms); GCSE = General Certificate of Secondary Education; GHQ = General Health Questionnaire (psychological distress); O-level = Ordinary level; PHQ = Patient Health Questionnaire (depressive symptoms); SE = standard error; SEN = special education needs; W1 = wave 1; W2 = wave 2

## Young caring and mental health

Table 2 shows the regression results. In crude, unadjusted models, young carers had higher levels of psychological distress (B = 0.71, 95% CI: 0.10, 1.32), depressive symptoms (B = 0.37, 95% CI: 0.21, 0.53), anxiety (B = 0.34, 95% CI: 0.18, 0.50) and higher odds of a reporting chronic mental illness (OR = 1.62, 95% CI: 1.30, 2.03). Estimates changed little after adjustment for covariates. However, after adjusting for wave 1 mental health, young carers no longer had higher levels of psychological distress at wave 2 (B = 0.34, 95% CI: -0.19, 0.87) but did still have higher levels of depressive symptoms (B = 0.20, 95% CI: 0.07, 0.33) and anxiety (B = 0.15, 95% CI: 0.02, 0.29).

**Table 2:** Associations between young caring and mental health

	GHQ (*n*=11,110)		PHQ (*n*=10,639)		GAD (*n*=10,628)		Chronic mental illness (*n*=9,571)	
	B	95% CI	B	95% CI	B	95% CI	OR	95% CI
Model 1 (crude)								
Young caring^a^	0.71	(0.10, 1.32)	0.37	(0.21, 0.53)	0.34	(0.18, 0.50)	1.62	(1.30, 2.03)
Model 2 (covariate adjusted)^b^								
Young caring	0.88	(0.26, 1.49)	0.36	(0.20, 0.52)	0.37	(0.21, 0.53)	1.69	(1.35, 2.12)
Model 3 (baseline mental health adjusted)^c^								
Young caring	0.34	(-0.19, 0.87)	0.20	(0.07, 0.33)	0.15	(0.02, 0.29)	NA	

^a^Reference for all models is non-carers

^b^Model 2 adjusts for school type, parental age, and parental education

^c^Model 3 = model 2 + adjustment for baseline (W1) mental health measure, except for chronic mental illness where this information was not available in W1

Abbreviations: GAD = Generalized Anxiety Disorder; GHQ = General Health Questionnaire; PHQ = Patient Health Questionnaire

### Inequalities in associations between young caring and mental health

Associations between young caring did not vary by gender, ethnicity, income, SEN status, nor parental composition (Table 3).

**Table 3:** *p* values from young caring inequality and support modification interactions

	GHQ	PHQ	GAD	Chronic mental illness
	*p* value	*p* value	*p* value	*p* value
Gender	0.748	0.261	0.756	0.719
Ethnicity	0.545	0.536	0.737	0.524
Income	0.469	0.881	0.755	0.604
SEN	0.767	0.804	0.695	0.923
Parental composition	0.970	0.791	0.414	0.183
School support	0.244	0.077	0.244	0.644
Social support	0.264	0.484	0.264	0.060
Peer support	0.418	0.008	0.418	0.116

Abbreviations: GAD = Generalized Anxiety Disorder; GHQ = General Health Questionnaire; PHQ = Patient Health Questionnaire; SEN = special educational needs

### Modification by peer, social and school support

**Fig. 1:**
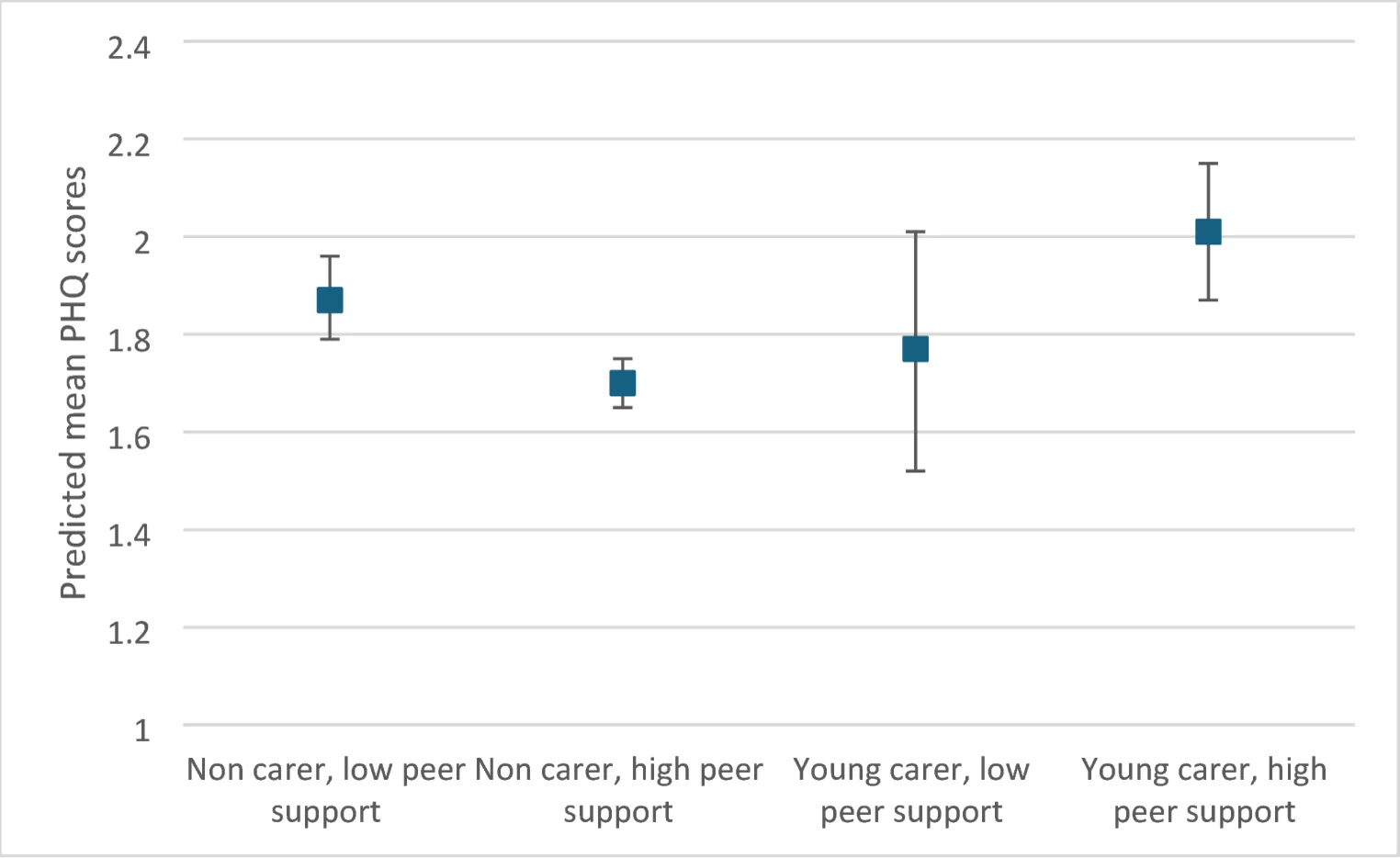
Predicted mean PHQ scores (95% Cis) by young caring and peer support

Associations between young caring and mental health varied by levels of support. Among non-carers, those with high peer support had fewer depressive symptoms than those with low peer support (non-carers, high peer support mean = 1.70, 95% CI: 1.65, 1.75; non-carers, low peer support mean = 1.87, 95% CI: 1.79, 1.96, Fig. 1). However, for young carers the opposite was true; young carers reporting high peer support reported the highest average number of depressive symptoms (mean 2.01, 95% CI: 1.87, 2.15; young carers, low peer support mean = 1.77, 95% CI: 1.52, 2.01). However, this difference in depressive symptoms was not statistically significant among young carers (contrast by levels of peer support in non-carers = -0.17, 95% CI: -0.27, -0.07; contrast by levels of peer support in young carers = 0.24, 95% CI: -0.04, 0.53).

**Fig. 2:**
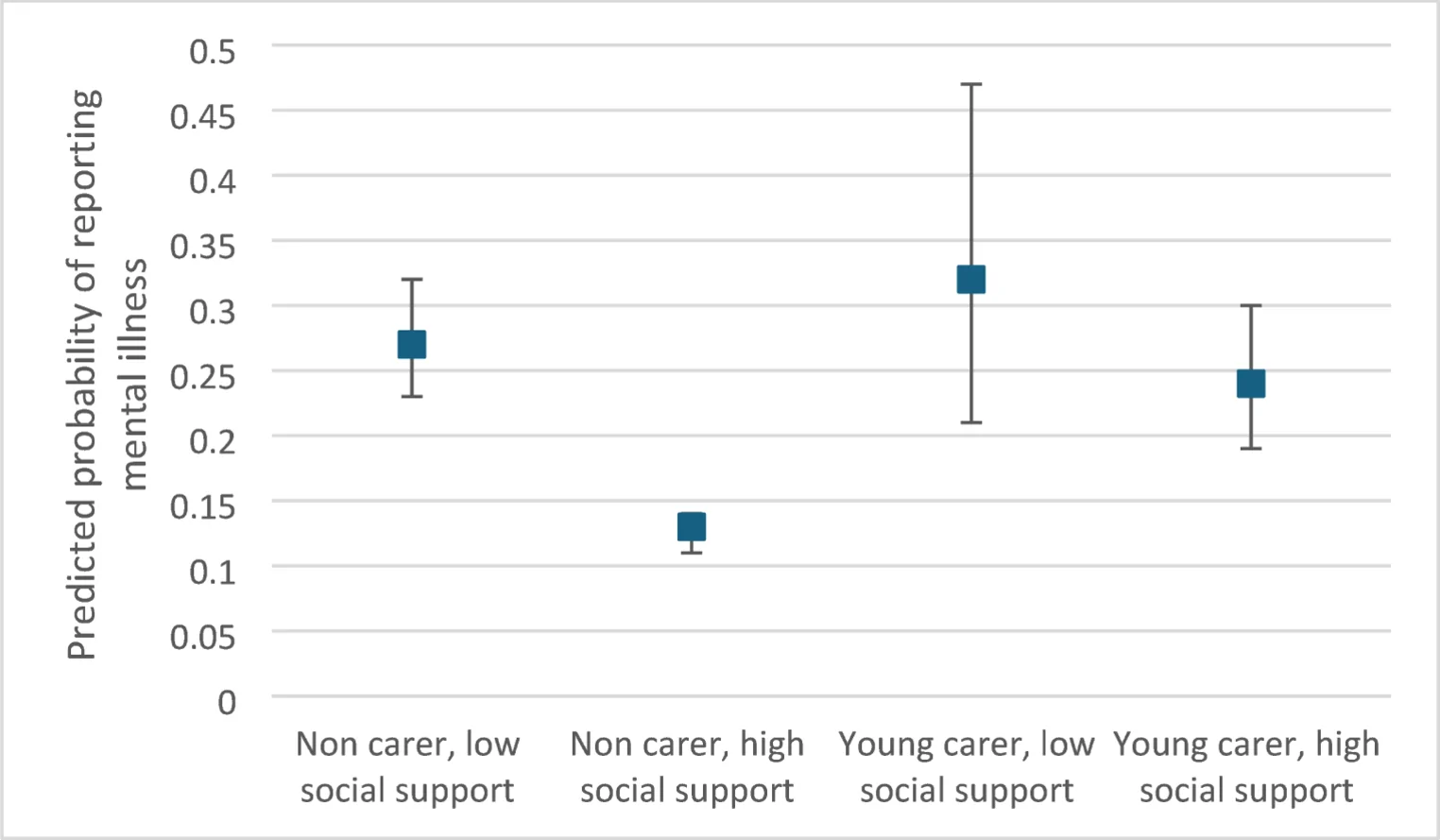
Predicted probabilities (95% Cis) of reporting a chronic mental illness by young caring and social support

There was additionally an indication of differences in the probability of reporting a chronic mental illness by young caring and social support (Fig. 2). There was a greater difference in the probability of reporting a chronic mental illness by levels of social support among non-carers than within carers. The probability of mental illness for non-carers with high social support was 0.13 (95% CI: 0.11,0.14) compared to 0.27 (95% CI: 0.23, 0.32) for non-carers with low social support. But for young carers there was no statistical difference observed in the probabilities of reporting a mental illness by social support level (young carer, low social support probability = 0.32, 95% CI: 0.21, 0.47; young carer, high social support probability = 0.24, 95% CI: 0.19, 0.30). Again this was confirmed by contrasts of predictive margins (levels of social support in non-carers contrast=-0.77, 95% CI: -0.98, -0.56; young carers, contrast=-0.30, 95% CI: -0.74, 0.14). No modification by perceived school support was observed.

### Sensitivity analysis

Findings from the complete case analyses (see Supplementary Information) were broadly similar to those in the imputed analyses presented here. In the complete cases we found that young carers had higher odds of reporting a chronic mental illness and reported more anxiety symptoms than non-carers. There were no differences observed between carers and non-carers on psychological distress and depressive symptoms after accounting for covariates and prior measures of mental health. In the complete case there was suggestion that young carers who reported good school support had more symptoms of anxiety compared to young carers with poor school support. There was also suggestion of modification by ethnicity in associations between young caring and chronic mental illness, but the estimates were unreliable and CIs very wide given the extent of missing information in ethnicity, so no firm conclusions could be made based on these data.

## Discussion

In this study, we demonstrated the mental health toll of young caring using a longitudinal design with a large, nationally representative sample of young people during the Covid pandemic. We showed that the mental health of young carers is considerably worse than their peers without caring responsibilities, with young carers being more likely to report a chronic mental illness and more depressive and anxiety symptoms. This finding aligns with the few other longitudinal studies of young carers’ mental health [8, 9, 17] and extends those findings to demonstrate the persistence of the mental health toll on young carers during and after the Covid pandemic. The associations between young caring attenuated somewhat when controlling for prior mental health, particularly for psychological distress, although we do not know whether psychological distress preceded young caring. Our analyses also went further than most previous studies to examine whether the associations between young caring and mental health varied by gender, household income, SEN status, parental composition or ethnicity to recognise that young carers are not a homogenous group. We anticipated that associations might be strongest in the most socially disadvantaged groups, but this was not the case. Prior studies have shown no gender differences in the relationships between young caring and mental health [35], and it appears that gender differences in the effects of caregiving emerge in early adulthood [36]. However, our study shows that the prevalence of young caring was higher in most of these social groups which means that these groups have a higher burden of young caring and mental health problems. This is consistent with the findings from other studies [2, 36]. 

Finally, we investigated whether support from peers, school and broader social networks make a difference. Previous research has shown that young carers want and benefit from someone trusted to talk to [26] and social support is one of the most important predictors of young carer’s adjustment.[37, 38] However young carers frequently report an unmet need in social support and large variations in the support available [26]. In our data, young carers with high levels of peer support had the highest levels of depressive symptoms, however this difference between young carers with high and low peer support was not statistically significant. Prior qualitative studies have found that young carers have strong connections with people outside of their family, including with peers[38] and frequently demonstrate high levels of life skills, including seeking help.[39] However, our study shows that peer support alone may not be enough to prevent mental health difficulties. [38] We additionally found that school and social support did not modify the relationship between young caring and mental health. Given that many young carers still remain hidden to schools (School Census returns in England show that 69% of schools reported having no young carers[40]) there is still a long way to go to improve young carer identification and support within school settings.

These findings highlight the importance of increasing societal awareness and empathy towards young carers. A recent qualitative study which investigated the types of support that young carers needed and valued [26], providing some guidance for support mechanisms that should be fostered to support young carers. These included formal and informal services. Young carers said that emotional support for the care recipient helped reduce the emotional burden placed on the young carer, that young carer groups were supportive and provided peers and trusted adults who understand their situation, and that mental health services offer flexible, patient and understanding support to the young carer (although waiting times for mental health services were long). In addition, broad school-based programmes also show promise (e.g. Young Carers in Schools scheme in England [41]), which aim to increase awareness and identification of young carers in the whole school community. Other potential interventions are those which directly aim to improve the mental health of young carers, such as the DNA-V model (Discover, Notice, Advisor and Values) [42], which includes cognitive behavioural therapy to support how the young person deals with challenging situations, thoughts, and feelings. Further, interventions that seek to reduce the extent of a young carer’s caring role (e.g. via funding for formal carers, respite care, increased social services input) have been cited by young carers as helpful to alleviate some of the mental health burden on young carers.[43] Successful interventions and support are likely to include multiple levels, including broader societal awareness through to more targeted, young carer-specific services.

### Strengths and Limitations

This study is not without its limitations. First, our study relies on self-reports of young caring and mental health. The latter does not include information on specific diagnoses, but instead uses broader population mental health instruments to pick up mental health problems at all levels of severity. Self-reports of caring rely on young people recognising the activities they undertake to support someone as “care work” and self-reported mental health chronic conditions also rely on the young person to recognise these or have been diagnosed. Related to this, we were unable to control for wave 1 chronic mental health conditions as this data was not available and hence the association may be over-estimated. Second, the SEN variable available only relates to SEN being given as the reason for school attendance during lockdowns. This is likely to underestimate the true prevalence of SEN, and more accurate information will become available once the NPD-COSMO linkage is available. Third, the peer, school and social support measures included do not tell us about the types or quality of support that is derived from these sources. Future studies should examine what it is, specifically, that helps prevent mental health problems in young carers. Fourth, the peer support measures only included items about friends and did not specifically include information about support from other young carers (a component of many young carer services). Fifth, these data were only on young people aged 16–18 and associations between young caring and mental health could not be examined at different ages. Sixth, it is possible that there are cultural and age-related differences in how the young caring question is interpreted and answered, which this study could not examine. Future research would benefit from co-designed and participatory approaches involving young carers to improve the relevance and sensitivity of survey questions and to guide qualitative follow-up. Seventh, while we have self-report care status we do not have information on who the young carer is providing care, how many hours of care they provide a week nor the activities that they are doing – these might be important for determining the extent to which mental health is affected [10]. Finally, it is possible that our inequality analyses were under-powered and these analyses should be repeated in a larger diverse population sample to aid our confidence in those findings. Regarding strengths, this study uses a large, representative, longitudinal study of young people during the Covid pandemic in England. This gives us high quality evidence on young carers’ mental health during the pandemic. We also applied multiple imputation to reduce the bias and loss of power attributable to missing data on caring and covariates. Young carers had more missing information than non-carers and multiple imputation is helpful to partially correct for any differential missingness.

## Conclusions

Young carers have poorer mental health than their peers without caring responsibilities. These associations did not differ by key axes of inequalities. Future research using larger, representative, well-powered samples are needed to clarify whether there are additional inequalities by these identities. More longitudinal research is needed on the mental health effects of young caring at other ages, including the youngest young carers (< 5 years) where there has been little focus to date. Inclusion of age-appropriate young caring questions in younger cohorts would help to facilitate this.

Multi-layered approaches are needed that improve awareness of young carers among the public, decision makers, educators and peers. In addition, interventions and services (e.g. social services input and formal care provision) which seek to reduce the caring responsibilities of young carers are also warranted.

## Electronic Supplementary Material

Below is the link to the electronic supplementary material.

Supplementary Material 1

## Data Availability

The data that support the findings of the study are openly available in the UK Data Service at [https://ukdataservice.ac.uk/](https:/ukdataservice.ac.uk) Reference number [https://doi.org/10.5255/UKDA-SN-9000-4](https:/doi.org/10.5255/UKDA-SN-9000-4).
