# Supplementary material for: Young carers’ mental health during and after the Covid-19 pandemic: variation by gender, ethnicity, income and peer, school or social support
Source: Soc Psychiatry Psychiatr Epidemiol. 2026 May 19;61(9):1855–65. doi: 10.1007/s00127-026-03111-6 (PMC13577991; doi:10.1007/s00127-026-03111-6)
Supplement: Supplementary file 1 [file 127_2026_3111_MOESM1_ESM.docx]

**Supplementary material**

**Complete case analysis**

**Associations between young caring and mental health**

|  | **GHQ (n=1,327)** | | **PHQ (n=1,323)** | | **GAD (n=1,328)** | | **Chronic mental illness (n=1,202)** | |
| --- | --- | --- | --- | --- | --- | --- | --- | --- |
|  | **B** | **95% CI** | **B** | **95% CI** | **B** | **95% CI** | **OR** | **95% CI** |
| Model 1 (crude) |  |  |  |  |  |  |  |  |
| Young caring^a^ | 1.50 | (-0.37, 3.38) | 0.48 | (0.04, 0.92) | 0.78 | (0.30, 1.26) | 2.04 | (1.21, 3.45) |
| Model 2 (covariate adjusted)^b^ |  |  |  |  |  |  |  |  |
| Young caring | 1.49 | (-0.41, 3.39) | 0.46 | (0.007, 0.92) | 0.81 | (0.31, 1.30) | 2.37 | (1.37, 4.11) |
| Model 3 (baseline mental health adjusted)^c^ |  |  |  |  |  |  |  |  |
| Young caring | 1.07 | (-0.39, 2.54) | 0.25 | (-0.12, 0.63) | 0.46 | (0.06, 0.85) | NA |  |

^a^Reference for all models is non-carers

^b^Model 2 adjusts for school type, parental age, and parental education

^c^Model 3 = model 2 + adjustment for baseline (W1) mental health measure, except for chronic mental illness where this information was not available in W1

Abbreviations: GAD = Generalized Anxiety Disorder; GHQ = General Health Questionnaire; PHQ = Patient Health Questionnaire

***p* values from young caring inequality and support modification interactions**

|  | **GHQ** | **PHQ** | **GAD** | **Chronic mental illness** |
| --- | --- | --- | --- | --- |
|  | ***p* value** | ***p* value** | ***p* value** | ***p* value** |
| Gender | 0.168 | 0.899 | 0.478 | 0.817 |
| Ethnicity | 0.389 | 0.381 | 0.085 | 0.035 |
| Income | 0.262 | 0.603 | 0.802 | 0.174 |
| SEN | 0.790 | 0.249 | 0.114 | 0.617 |
| Parental composition | 0.311 | 0.750 | 0.418 | 0.552 |
| School support | 0.146 | 0.282 | 0.043 | 0.925 |
| Social support | 0.895 | 0.177 | 0.995 | 0.213 |
| Peer support | 0.563 | 0.583 | 0.574 | 0.449 |

Abbreviations: GAD = Generalized Anxiety Disorder; GHQ = General Health Questionnaire; PHQ = Patient Health Questionnaire; SEN = special educational needs

**Association between young caring and GAD stratified by school support**

|  | **GAD** | |
| --- | --- | --- |
|  | **B** | **95% CI** |
| Young caring + good school support | 0.85 | 0.26, 1.44 |
| Young caring + poor school support | 0.13 | -0.34, 0.60 |

Each row is in comparison to non-carer with the same level of school support

**Association between young caring and chronic mental illness stratified by ethnicity**

|  | **Chronic mental illness** | |
| --- | --- | --- |
|  | **OR** | **95% CI** |
| Young caring + White ethnicity | 2.36 | 1.27, 4.38 |
| Young caring + Asian/Asian British ethnicity | 11.73 | 0.91, 151.70 |
| Young caring + Black ethnicity | 0.21 | 0.0001, 4.02 |
| Young caring + Mixed/other ethnicity | 5.83 | 0.68, 50.35 |

Each row is in comparison to a non-carer within the same ethnicity category
